# CryGetter: a tool to automate retrieval and analysis of Cry protein data

**DOI:** 10.1186/s12859-016-1207-2

**Published:** 2016-08-30

**Authors:** David Buzatto, Suzelei de Castro França, Sônia Marli Zingaretti

**Affiliations:** 1 Instituto Federal de Educação, Ciência e Tecnologia de São Paulo – IFSP, Câmpus São João da Boa Vista, Acesso Dr. João Batista Merlin, s/n, Jardim Itália, São João da Boa Vista, 13872-551 SP Brazil; 2 Universidade de Ribeirão Preto – UNAERP, Av. Costábile Romano, 2201, Ribeirânia, Ribeirão Preto, 14096-000 SP Brazil

**Keywords:** Cry protein, Protein analysis, Sequence alignment, Automatic data retrieval

## Abstract

**Background:**

For many years, the use of chemical agents to control crop pests has been degrading the environment, bringing problems to humans and all living things. An alternative to deal with the pests is the use of biopesticides, biological agents capable of controlling these harmful organisms. One kind of biopesticide is *Bacillus thuringiensis*, a Gram-positive bacterium that synthesizes a protein that, when ingested by the pests, kills them and does not harm other species.

**Results:**

Since the economical importance of *Bacillus thuringiensis* and its proteins significance, this work presents a software tool, called CryGetter, that is capable of retrieving data related to these proteins, store it and present it in a user friendly manner. The tool also aims to align the protein sequences and generate reports containing some statistical data concerning the alignments that were made.

**Conclusions:**

CryGetter was created to help researchers of *Bacillus thuringiensis* and its proteins to speed up their data retrieval and analysis, allowing them to generate more accurate results. In this sense, the tool circumvents the error prone task of manually getting all the necessary data and processing them in various software systems to get the same result as CryGetter gets in a unique semiautomatic environment.

## Background

The biopesticides produced by the *Bacillus thuringiensis* (*Bt*) bacterium are a viable alternative for crop pest control using chemical pesticides [1, 2] without the collateral effects of environment contamination, since the toxins synthesized by *Bt* have little effect on non-target insects and vertebrates like birds and mammals [3–7]. The *Bt* is a bacterium present in the soil and produces a protein called Crystal protein (Cry protein) during the sporulation phase. Such protein is lethal to various insect orders [8], including *Coleoptera*, *Lepidoptera* and *Diptera*.

**Fig. 1:**
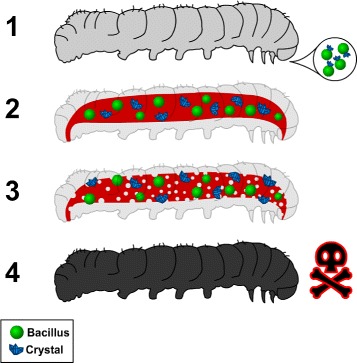
Cry protein mode of action (adapted from [50])

To date, about 600 genes that encode the Crystal protein from different isolates were identified [1]. These genes are sorted according to the insect order to which the protein is toxic to [9–11]. Once ingested (Fig. 1, sections 1 and 2), the Cry protein acts in the insect gut by opening pores in the intestinal membrane (Fig. 1, section 3), which causes the death of larvae due to starvation and/or bacterial infection (Fig. 1, section 4). There are two hypotheses regarding how these proteins act [12, 13]. Both propose the interaction of protein receptors present in the insect’s gut; on both models, the cadherin proteins (CADR) are the first receptors to be activated. This interaction between the Cry proteins and their receptors occurs in some regions of the gene known as Domains I, II and III. Domain I is involved in the process of membrane insertion and pore formation. Domains II and III are both related to receptor recognition and binding. The third domain is also related to the role of pore formation [11].

Regarding *Lepidoptera*, at least four types of protein receptors are involved in the binding process of proteins to Brush Border Membrane Vesicles (BBMV), beginning the process of pore formation in different *Lepidoptera* larvae: a cadherin-like protein (CADR), a glycosylphosphatidyl-inositol (GPI)-anchored aminopeptidase-N (APN), a GPI-anchored alkaline phosphatase (ALP) and a 270kDa glycoconjugate [14]. The protein encoded by the cry genes from some strains of *Bt* are specific to certain insect orders, while others do not exhibit this specificity and can act on two or more different orders. The reason for this specificity is still unknown, but amino acid changes in this region have been associated with toxicity. Tiewsiri and Angsuthanasombat [15] made the substitution by an Alanine (A) in four highly conserved aromatic residues (^242^W^244^, ^245^F^247^, ^248^Y^250^ and ^263^F^265^) in Cry4B gene, that has a toxin that attacks insects of the *Diptera* order. This resulted in a decrease of toxicity to the mosquito *Stegomyia aegypti*. In recent decades, several plants of different species have been transformed with genes of *Bt* to be commercialized (*Zea mays*, *Gossypium hirsutum*, *Glycine canescens*, *Oryza sativa*, etc.). The pattern recognition in the amino acid sequence of these proteins, that may be associated with specificity, could facilitate the use of these genes in the generation of new transgenic plants resistant to different crop pests, as well as the construction of *Bt* pyramided plants [16, 17], which is a reality nowadays.

There are many software tools capable of analyzing protein sequences like MEGA 6 (Molecular Evolutionary Genetics Analysis) [18, 19], MacClade [20] and Geneious [21–23]. MEGA 6 is capable of storing and processing protein sequences in order to align them, generate phylogeny trees and perform other calculations. It can also work with DNA sequences. The tool can even search online databases for data of interest that can be downloaded free of charge. Like MEGA 6, MacClade is also capable of analyzing DNA sequences to construct their phylogeny trees, allowing the user to identify molecular evolution among the tested gens, but it is only available for Mac OS. Geneious is a more complete tool, since it supports the features of MEGA 6 and MacClade in addition to having more options for protein and DNA analysis. It can also be extended by the use of plug-ins [24, 25]; however it is a paid tool.

In order to support the study of Cry proteins, we created a specialized and open-source software tool that is capable of compile the data of Cry proteins in a central repository, allowing the retrieval of data related to each protein such as their primary structures, three-dimensional models (PDB files), related works, etc., allowing the manipulation of such data in alignment algorithms and generation of reports related to the alignments. This tool, called CryGetter, is presented and detailed in this work.

## Implementation

**Fig. 2:**
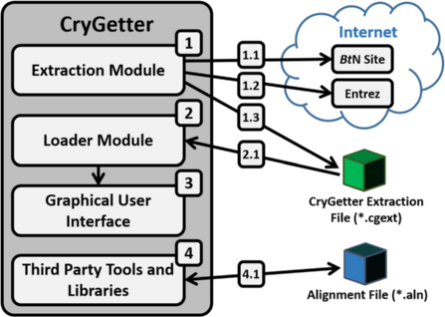
CryGetter application architecture

To implement CryGetter, we used the Java SE (Standard Edition) Platform, version 8 [26] and other third party tools and libraries to perform some tasks like protein sequence alignment, alignment visualization (using MView [27] tool) and molecule rendering (using BioJava [28] and Jmol [29] libraries). In addition, CryGetter uses the data of the full toxin list of *Bacillus thuringiensis* Toxin Nomenclature website [30] and Entrez [31] (Global Query Cross-Database Search System) service of NCBI (National Center for Biotechnology Information) for automatic retrieval of protein data and Cry protein models available at the PDB [32, 33] (Protein Data Bank) and the PMDB [34, 35] (Protein Model Database). The application architecture is shown in Fig. 2 and each section of this architecture is explained below.

**Fig. 3:**
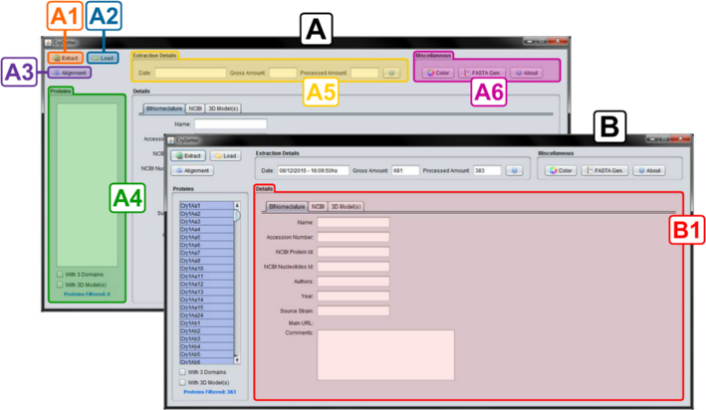
CryGetter main interface and its features

The “Extraction Module”, highlighted in Fig. 2 using the number 1, represents the data extraction feature of CryGetter that is performed by this module of the tool and can be executed pressing the button “Extract” highlighted in section A1 of Fig. 3.

When pressed, this button triggers the execution of the extraction module that executes four steps:

1. **BtNomenclature site data extraction:** First, the extraction module retrieves the data of a particular HTML (HyperText Markup Language) file ^1^ of the BtNomenclature website (arrow 1.1 in Fig. 2). This file contains a HTML table with all Cry proteins that already studied and cataloged by the website owner;
2. **Cry protein data preprocessing:** The data from the raw HTML file gathered in the previous step is then processed using the jsoup [36] library to create a linked list of an ADT (Abstract Data Type) called “CryToxin”, which contains only Cry proteins entries (from BtNomenclature website) that has a NCBI hyperlink related to a protein sequence. This ADT and its composition are presented in the UML (Unified Modeling Language) class diagram showed in Fig. 4. This class composition was modeled this way because we need to serialize this data from Java objects to XML (Extensible Markup Language) and deserialize it from XML to Java objects;

**Fig. 4:**
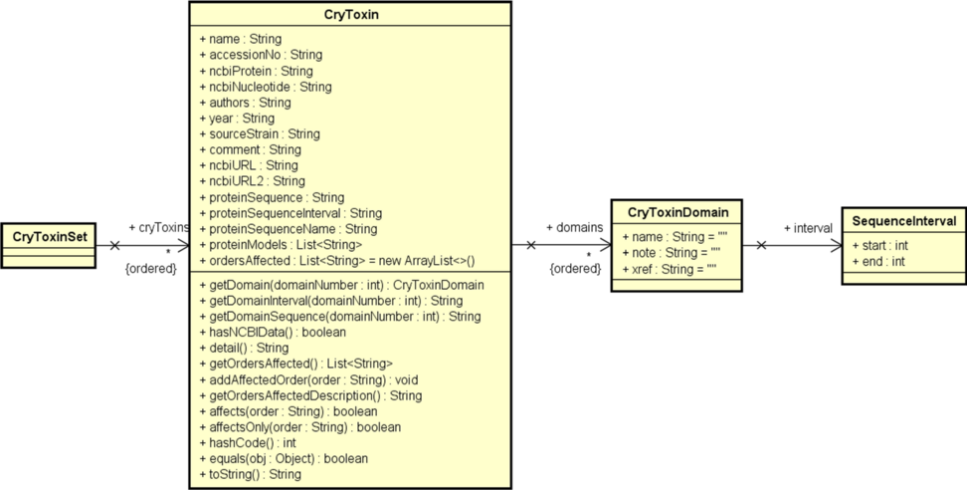
Class diagram for CryToxin ADT and its composition
3. **NCBI data extraction:** Using the Entrez service (arrow 1.2 in Fig. 2), all proteins GI numbers of each Cry protein entry that was acquired in the previous steps are used to retrieve all data related to each protein. In this case, the URL (Uniform Resource Locator) used to access the Entrez service is https://eutils.ncbi.nlm.nih.gov/entrez/eutils/efetch.fcgiand some URL parameters need to be passed:
  **tool:** the name of the tool that is accessing the Entrez service. In this case, “crygetter”;
  **email:** e-mail of the responsible for the tool. In this case, “davidbuzatto@ifsp.edu.br”;
  **db:** the database that is being accessed. In this case “protein”;
  **retmode:** the type of data that will be returned by the service. In this case “xml”;
  **id:** the set of ids, separated by commas, that represents the proteins. In this case, all Cry proteins GI that were retrieved in the previous steps;
  **Obs:** more details about the Entrez HTTP (Hypertext Transfer Protocol) interface can be found in its documentation [31].
  The result of the request to the Entrez service, in XML format, is processed by the tool, generating a set of temporary files that will later be used in the next step. To process these generated files, the tool needs to parse the returned XML and generate a object composition in memory. To do this, a class composition that reflects the XML structure was created and is shown in Fig. 5. For the sake of simplicity, in this diagram we do not show the class compartments for attributes and operations. This composition is used by the tool to store the result of the XML deserialization, allowing the data to be processed and used to further complement the CryToxin ADT data;

**Fig. 5:**
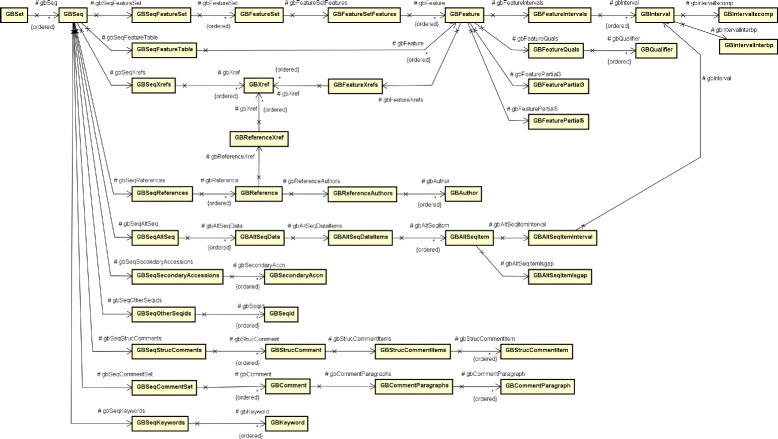
Class diagram for the XML structure defined by Entrez service return
4. **CryGetter extraction data file generation:** The last step comprises the creation of a file that will be saved by the user (arrow 1.3 in Fig. 2). This file will contain all the data that was obtained and it will be able to be opened by anyone who has the tool. To process the stored data we used two XML parsing libraries: SimpleXML [37] for serialization and deserialization of CryToxin ADT and JAXB [38] (Java Architecture for XML Binding) for serialization and deserialization of Entrez XML data. We chose SimpleXML to process the tool specific XML data and JAXB to manipulate the Entrez return, since the class composition for Entrez was automatically generated by the JAXB compiler, available by default in any JDK (Java Development Kit), using the Entrez DTD ^2^ (Document Type Definition).
  To open an extraction file, the user must click on the “Load” button highlighted in section A2 of Fig. 3. By clicking on this button, an open dialog box will appear and the user will choose the file that s/he wants to open. When a file is chosen, the “Loader Module” (highlighted in Fig. 2 using the number 2) will execute, unpacking the file (arrow 2.1 in Fig. 2) and presenting its contents in the main interface (represented in Fig. 2 by the “Graphical User Interface” section (highlighted by the number 3) of the tool as showed in section B of Fig. 3.

As result, all Cry protein data is loaded into the tool and shown in the left list (the empty list is highlighted in section A4 of Fig. 3 and the filled list is presented in section B of the same Figure), allowing the user to select the protein that s/he wants to perform tasks like data visualization (section B1 of Fig. 3), protein alignment (accessed by the button “Alignment”, presented in section A3 of Fig. 3), protein alignment visualization, protein analysis and FASTA file generation (section A6 of Fig. 3). All these tasks use one or more third party libraries or third party tools (represented in Fig. 2 by the “Third Party Tools and Libraries” section, highlighted by the number 4) and each task is explained hereafter.

### Cry protein data visualization

**Fig. 6:**
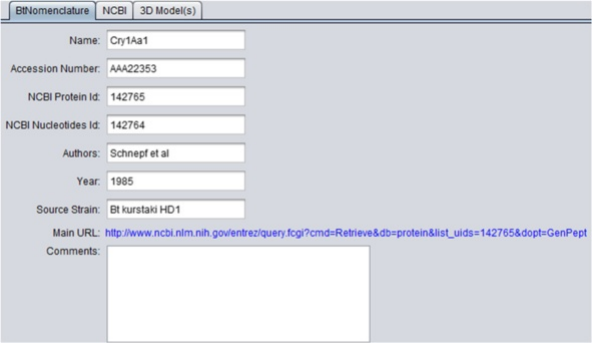
BtNomenclature details tab
- **NCBI:** It presents the data collected in NCBI, through Entrez service. This tab is shown in Fig. 7 and the details are divided into three tabs:
  - **Main:** The main data related to the selected protein (section A of Fig. 7);

The main functionality related to Cry proteins is data visualization. When the user selects a protein in the list on the left, like Cry1Aa1, all of its related data are shown in tabs located on the right, represented in Fig. 3 by section B1. There are some tabs organized in a hierarchical mode:

- **BtNomenclature:** It presents the data collected in the BtNomenclature website. This tab is shown in Fig. 6;

**Fig. 7:**
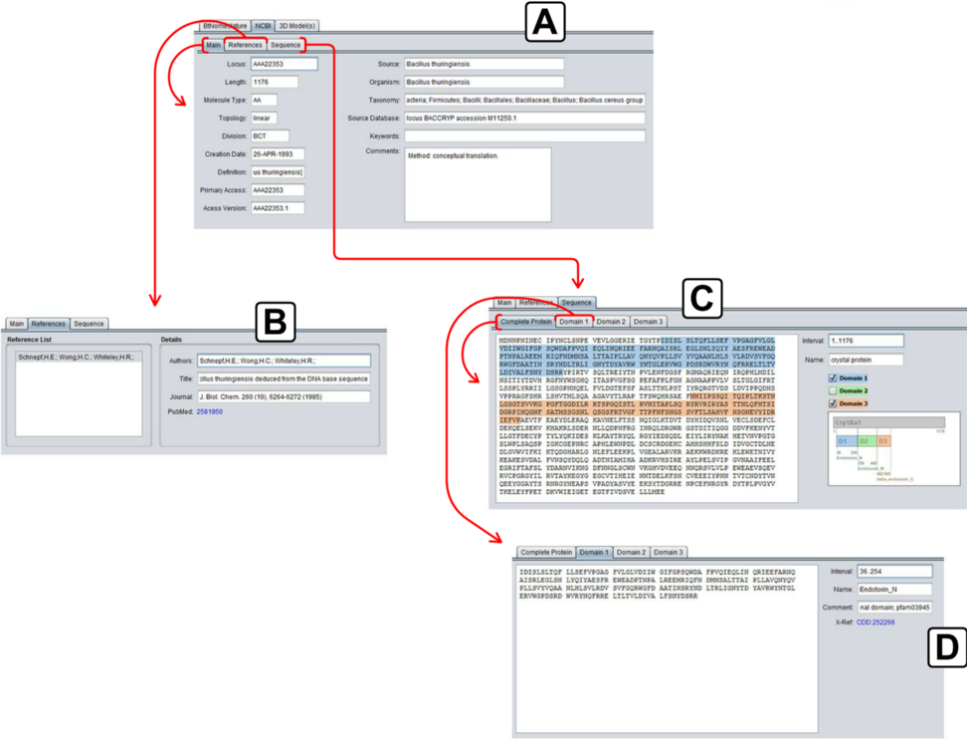
NCBI details tab. **a**) Main tab of NCBI details tab; **b**) References tab of NCBI details tab; **c**) Sequence tab of NCBI details tab; **d**) Domain 1 tab of Sequence tab
  - **References:** The set of references related to that protein (section B of Fig. 7);
  - **Sequence:** The amino acid sequence of the selected protein (primary structure), divided into three domains (if these domains are present in the NCBI data) and a diagram showing the position of the domains inside the entire sequence. This tab is then divided in four tabs:
    – **Complete Protein:** The complete amino acid sequence of the
    protein (section C of Fig. 7);
    – **Domain 1, 2 and 3:** Three tabs containing the data of each domain
    of the protein (Domain 1 shown in section D of Fig. 7).
- **3D Model(s):** In this tab, the 3D models (if they exist) of the protein are listed (Fig. 8), so the user can use them in some third party visualization tool like VMD [39, 40] (Visual Molecular Dynamics), Swiss PDB Viewer [41, 42] and/or PyMol [43] to visualize and/or process them. It’s important to emphasize that, nowadays, there are 22 experimental models, related to 18 different Cry proteins, deposited in PDB and PMDB and all these models are available in CryGetter. The Table 1 shows the proteins that have models available in PDB and PMDB along with their related works. In this table we also list some proteins that have papers reporting the creation of their models, but these models are note available neither in PDB nor in PMDB. It is also important to note that CryGetter isn’t able to generate new models by using some protein structure prediction software.

**Fig. 8:**
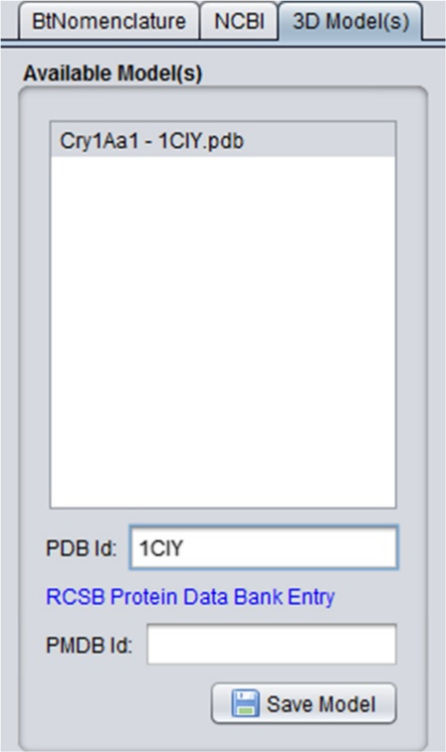
3D models details tab

**Table 1:** Cry proteins models available in PDB and PMDB and others just described in literature

Protein	Affected order(s)	Model Id ^*a*^	Reference(s)	Obs.
Cry1Aa1	*Diptera*,	1CIY	[51, 52]	
	*Lepdoptera* and			
	*Gastropoda*			
Cry1Ab16	*Diptera*,	unavailable	[53]	There isn’t a deposited model
	*Lepdoptera* and			
	*Gastropoda*			
Cry1Ab19	*Diptera*,	unavailable	[54]	There isn’t a deposited model
	*Lepdoptera* and			
	*Gastropoda*			
Cry1Ac1	*Diptera*,	4ARX	Not yet published	
	*Lepdoptera* and	4ARY	Not yet published	
	*Gastropoda*	4W8J	[55]	
Cry1Ld	*Lepdoptera*	unavailable	[56]	There isn’t a deposited model
Cry2Aa1	*Diptera*,	1I5P	[57]	
	*Hemiptera* and			
	*Lepdoptera*			
Cry3A	*Coleoptera*,	1DLC	[58]	
	*Hemiptera* and			
	*Hymenoptera*			
Cry3Aa1	*Coleoptera*,	4QX0	[59]	
	*Hemiptera* and	4QX1		
	*Hymenoptera*	4QX2		
Cry3Bb1	*Coleoptera*	1JI6	[60]	
Cry4Aa1	*Diptera*	2C9K	[61, 62]	
Cry4Ba1	Diptera	1W99	[63]	
		4MOA	Not yet published	
Cry5Aa1	*Hymenoptera* and	PM0074964	[64]	
	*Rhabditida*			
Cry5B	*Rhabditida*	4D8M	[65]	
Cry5Ba1	*Rhabditida*	PM0075036	[66]	
Cry6Aa	*Rhabditida*	5J66	Not yet published	Pore formation
Cry23Aa1	*Coleoptera*	4RHZ	Not yet published	Binary protein complex
Cry37Aa1				
Cry8Ea1	*Coleoptera*	3EB7	[67]	
Cry11Bb1	*Diptera*	unavailable	[68]	There isn’t a deposited model
Cry30Ca2	*Diptera*	unavailable	[69]	There isn’t a deposited model
Cry34Ab1	*Coleoptera*	4JOX	[70]	
Cry35Ab1	*Coleoptera*	4JP0	[70]	
Cry51Aa1	*Coleoptera* and	4PKM	[71]	
	*Hemiptera*			

^a^The model ids with 4 characters are from PDB while the model ids with 9 characters are from PMDB

Other features of the tool’s main interface are the extraction details (the date of extraction, gross amount of gathered proteins and the processed amount of the proteins, highlighted in the section A5 of Fig. 3), the color change dialog (to change the color of the presented proteins inside the protein list) and the “FASTA Gen.” button, used to generate a set of FASTA files of all proteins (section A6 of Fig. 3). For each protein, four files are created, one with the complete sequence and one for each of the three domains. In addition of these features, the tool can perform protein alignment for further analysis. This functionality and its sub features are presented in the next section.

### Cry protein alignment

**Fig. 9:**
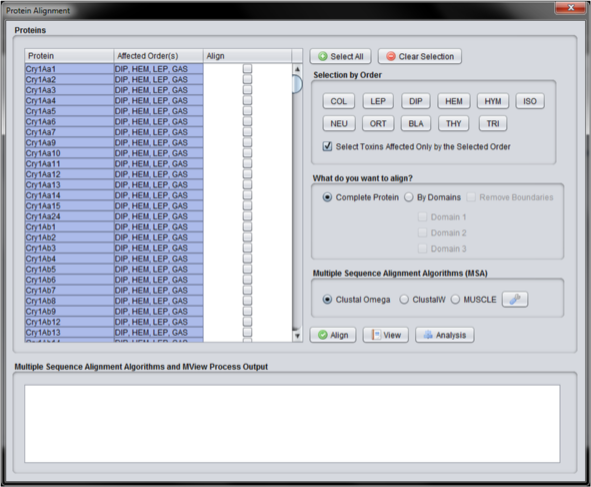
Protein alignment interface

The “Cry Protein Alignment” interface can be accessed through the “Alignment” button located on the top left corner of the CryGetter main interface (section A3 of Fig. 3) and it is shown in Fig. 9.

**Fig. 10:**
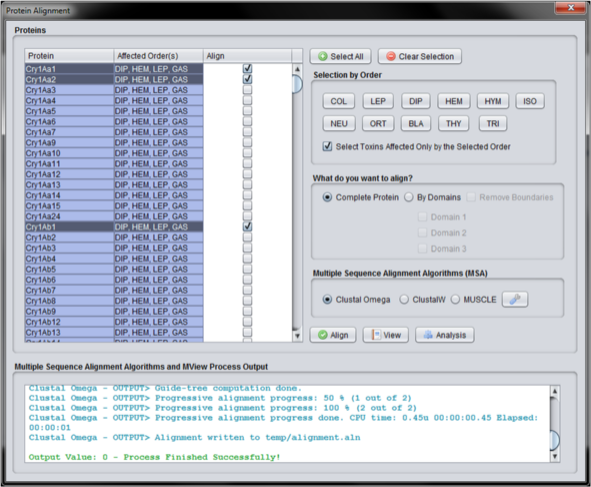
Alignment result of Cry1Aa1, Cry1Aa2 and Cry1Ab1

In this interface, the user can perform a set of protein alignment tasks with the Cry proteins, generating and/or loading alignment data files (arrow 4.1 in Fig. 2). There are some filters that can be used, such as selecting the proteins by the affected orders. The orders affected by each protein are stored in a XLSX (Office Open XML SpreadsheetML File Format) file that is processed by the Apache POI [44] library. The user can define what sections of the selected proteins s/he wants to align and the MSA (Multiple Sequence Alignment) algorithm that should perform the alignment task. In the current version of the tool, three algorithms are available: Clustal Omega [45], Clustal W [45, 46] and MUSCLE [47]. These three algorithms, executed by external tools, can also be parametrized in CryGetter. As an example, in Fig. 10 we show the result of the alignment of Cry1Aa1, Cry1Aa2 and Cry1Ab1 after executing the Clustal Omega algorithm. When the algorithm finishes its execution, a dialog box is shown to the user, allowing s/he to save the alignment data, which can now be visualized or analyzed.

### Alignment results processing

**Fig. 11:**
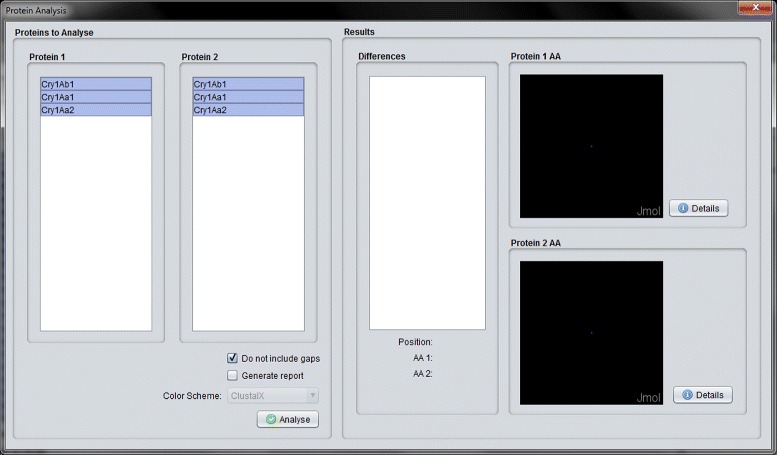
Protein analysis interface with the result of the alignment of Cry1Aa1, Cry1Aa2 and Cry1Ab1

When two or more Cry proteins are aligned, CryGetter can perform an analysis on the alignment result. When clicking the “Analysis” button (Fig. 10), a dialog appears and the user is asked to choose the alignment data file that was generated. By doing this, the program reads the data and shows the “Protein Analysis” interface, as shown in Fig. 11.

**Fig. 12:**
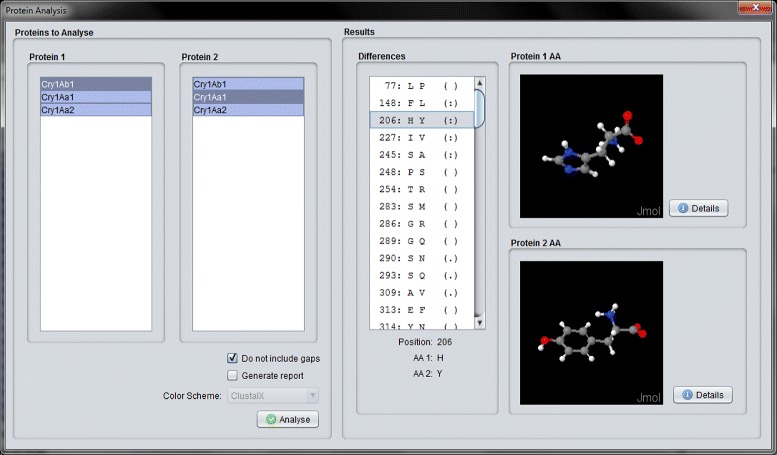
Comparison between Cry1Ab1 and Cry1Aa1

In this interface, the user can analyze the alignment by choosing two different proteins and clicking on the “Analyse” button. Doing so, the program will identify the differences between the selected proteins and show them in the differences list. In the example shown in Fig. 12, the differences between the proteins at location 206 is presented. In this case, Cry1Ab1 has an ^205^H^207^ (Histidine) amino acid and Cry1Aa1 has a ^205^Y^207^ (Tyrosine) amino acid. The user can even generate a report that summarizes the alignment data. This feature is presented in the next section, where an experiment comprising the analysis of two different Cry proteins was conducted.

## Results and discussion

CryGetter is currently being used as a support tool for two works related to Cry proteins structure analysis. The tool is proving to be useful, since it can automatize the data extraction task and perform a sort of functions related to proteins, drastically reducing possible errors in manual data collection of online sources, since if such task was being done manually, the chance of human error would be enormous, because the process of copy/paste large quantities of data, for example, of protein sequences available through a NCBI web-page to a specific tool, would probably add undesirable and difficult to detect errors like, for example, data truncation, unless all sequences are constantly reviewed. In the next subsection we present a study case of the use of the tool.

### Using CryGetter to Analyse two different order specific cry proteins

For this study, two Cry proteins were chosen to be analyzed based in their order specificity:

- **Cry1Aa1:** this Cry protein affects mainly the *Diptera* order;
- **Cry3Aa1:** the *Coleoptera* order is affected by this type of Cry protein.

**Fig. 13:**
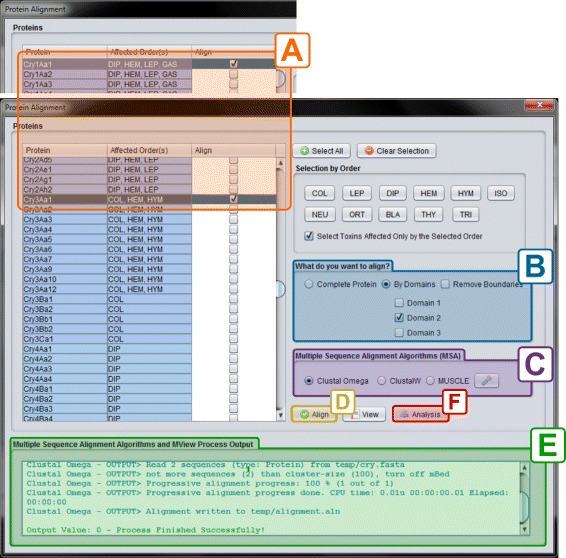
Preparing the alignment of Cry1Aa1 and Cry3Aa1. **a**) Selection of Cry proteins to align; **b**) Alignment constraints; **c**) Selection of MSA algorithm; **d**) Align button; **e**) MSA algorithms output; **f**) Analysis button

After loading a generated data package in CryGetter, the “Alignment” button (presented in section A3 of Fig. 3) was clicked, aiming to show the “Alignment Interface”. Since we wanted to analyse Cry1Aa1 and Cry3Aa1, these two protein were selected (clicking on the check-box of the “Align” column) in the protein list (highlighted by the section A of Fig. 13). After the selection, in section B of Fig. 13 we chose to align only the Domain 2 of both proteins, since this domain is mainly responsible for receptor recognition [11] and we wanted to study what is the difference between these two proteins that made their specificity occur. In section C of Fig. 13, the Clustal Omega MSA was selected and finally the “Align” button (section D of Fig. 13) was clicked on. Doing this, the Clustal Omega executable performed the alignment of the selected sequences and a file with the result was saved on disk. The execution output of the selected MSA is shown in section E of Fig. 13.

**Fig. 14:**
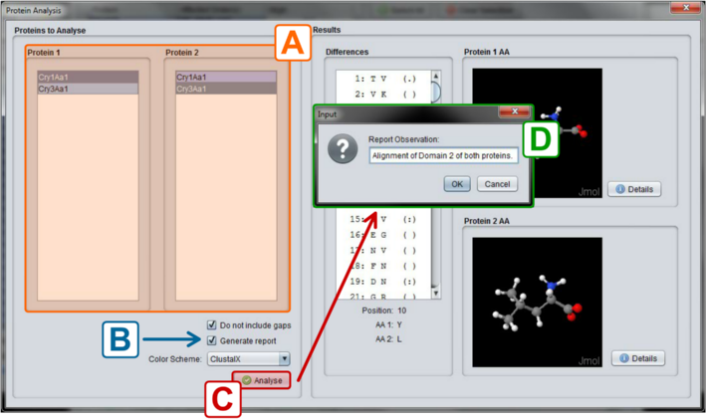
Analysing the alignment of Domain 2 Cry1Aa1 and Cry3Aa1. **a**) Lists of proteins to analyse; **b**) Analysis options; **c**) Analysis button; **d**) Report Observation dialog

Now, we wanted to analyze the alignment. To do this, we clicked on the button “Analysis” (section F of Fig. 13). After clicking on it, CryGetter required an alignment file. We chose the previously generated alignment file. By doing this, the “Protein Analysis Interface” was shown. In this interface, we chose the two proteins that we wanted to analyze. This selection is shown in section A of Fig. 14. CryGetter was able to infer some results based on the alignment. To do this, we selected the “Generate report” check-box (section B of Fig. 14). Finally, we hit the “Analyse” button (section C of of Fig. 14). A dialog (section D of Fig. 14) was presented, allowing the user to insert some textual observation. In this case, we wrote that these results were related to the alignment of the Domain 2 of both proteins. Clicking on OK, a report was then generated.

**Fig. 15:**
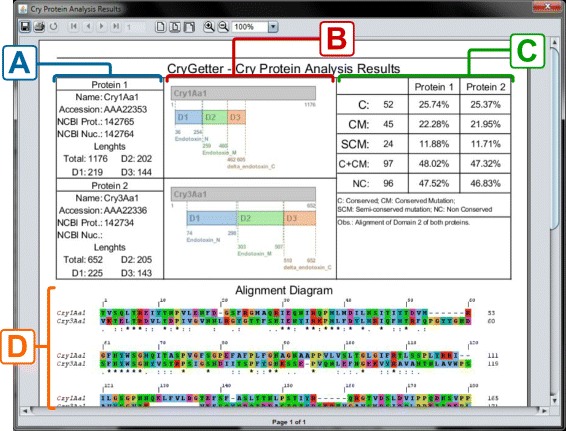
Analysis report. **a**) Protein data; **b**) Structure diagram; **c**) Report statistics; **d**) Alignment diagram
- CM: the amount of conserved mutations;
- SCM: how many semi-conserved mutations;
- C+CM: the sum of C and CM;
- NC: the amount of non conserved residues.

This report, created using the JasperReports library [48] and presented in Fig. 15, contained the data of both proteins, presented in section A of Fig. 15. These details comprised the name, accession number, protein id and nucleotide id of the protein, besides the total length of the protein sequence and the size of the three domains. In section B of Fig. 15. a structure diagram of both proteins is presented, showing the disposition of the domains inside the complete sequence, their boundaries and names. In section C of Fig. 15, some statistics are shown:

- C: the amount of conserved residues;

The percentages shown in columns Protein 1 and Protein 2 are related to the amount of each item versus the total number of residues. For example, C is equal to 52, that is 25.74 % of 202 residues of Domain 2 in Protein 1 and 25.37 % of 205 residues of Domain 2 in Protein 2. The total number of residues that are used in these calculations is equal to the size of the sequence that it was aligned to. In this example, we chose to align only Domain 2, that has 202 and 205 residues in Cry1Aa1 and Cry3Aa1 respectively. Finally, in section D of Fig. 15, a complete alignment diagram of the two proteins is shown.

The analysis performed by the tool can be used as a starting point to further analyze two Cry proteins. The importance of the tool can be noted, since it simplifies all the process of getting the sequences of interest, aligning them and performing the preliminary inspections in the alignment result.

## Conclusions

In this work we presented CryGetter, a tool that aggregates Cry protein data, helping researchers of *Bacillus thuringiensis* and its Crystal proteins to deal with this data and allowing them to get all the relevant information for their work in a faster way compared to a manual protein data collection. Since the tool executes data retrieval and can perform automatic analysis os the protein alignments, it allows their users to generate more accurate results, since using it may prevent the error prone task of manually getting all the necessary data and processing them in various software to get the same result that the tool can generate in a unique automatic environment. The development of the tool is also important since these proteins play a significant role in the agro-industry. We hope CryGetter can help the researcher community to improve and accelerate their work with Cry proteins, getting preliminary results faster. As a future work, we will work in the generalization of the tool, allowing the users to extrapolate its functionality related to the data retrieval, enabling them to get data from different online data-sources.

## Availability and requirements

**Project name:** CryGetter**Project home page:**http://davidbuzatto.github.io/CryGetter**Operating system(s):** Microsoft Windows^®;^ 7 or above**Programming language:** Java**Other requirements:** Java Runtime Environment (JRE) 8 [26] and Perl runtime [49] for MView [27]**License:** GNU General Public License v3.0**Any restrictions to use by non-academics:** None

## Abbreviations

ADT: Abstract data type
ALP: Alkaline phosphatase
APN: Aminopeptidase-N
BBMV: Brush border membrane vesicles
Bt: Bacillus thuringiensis
CADR: cadherin-like protein
DTD: Document Type definition
GPI: Glycosylphosphatidyl-inositol
HTML: HyperText markup language
HTTP: Hypertext transfer protocol
JAXB: Java architecture for xml binding
JDK: Java development kit
JRE: Java runtime environment
MEGA: Molecular evolutionary genetics analysis
MSA: Multiple sequence alignment
NCBI: National center for biotechnology information
PDB: Protein data bank
PMDB: Protein model database
SE: Standard edition
UML: Unified modeling language
URL: Uniform resource locator
VMD: Visual molecular dynamics
XLSX: Office open XML SpreadsheetML file format
XML: Extensible markup language
